# The Care Need Index for Child Health Services in Sweden Showed Relative Stability From 2022 to 2024

**DOI:** 10.1111/apa.70694

**Published:** 2026-07-16

**Authors:** Mattias Wennergren, Anna Grimby‐Ekman, Rajna Knez, Sofia Dalemo, Anna Fäldt

**Affiliations:** ^1^ School of Public Health and Community Medicine, Institute of Medicine Sahlgrenska Academy, University of Gothenburg Gothenburg Sweden; ^2^ Research, Education, Development and Innovation, Primary Health Care Region Västra Götaland Gothenburg Sweden; ^3^ Department of Child Health Services Region Västra Götaland Gothenburg Sweden; ^4^ Research, Education, Development, and Innovation Unit Skaraborg Hospital Skövde Sweden; ^5^ Institute of Neuroscience and Physiology Sahlgrenska Academy, University of Gothenburg Gothenburg Sweden; ^6^ School of Health Sciences University of Skövde Skövde Sweden; ^7^ Child Health and Parenting (CHAP), Department of Public Health and Caring Sciences Uppsala University Uppsala Sweden

The allocation of Swedish primary healthcare resources is partly based on the local sociodemographic characteristics of the enrolled population [1]. Sociodemographic deprivation may influence the way healthcare is used and the associated costs. However, Swedish child health services have seen differences in usage and health outcomes across sociodemographic contexts. In some cases, more deprived populations have received insufficient access to services, while less deprived populations have received more [2]. Indexes that incorporate sociodemographic risk factors, like the Child Adjusted Care Need Index [3], aim to support equitable resource allocations by directing additional resources to populations with greater levels of sociodemographic disadvantage. Because all Swedish citizens are free to choose their primary care provider (Healthcare Centre), and these units are often responsible for the Child Health Services [1, 4], we have used the term Child Healthcare Centre throughout the paper regardless of organizational structure. The distribution of healthcare needs and sociodemographic characteristics varies across centres and may shift over time. Our aim was to investigate whether the Child Adjusted Care Need Index for Child Healthcare Centres indicated any major changes between 2022 and 2024.

Child Adjusted Care Need Index scores, for all Child Healthcare Centres in Sweden, were retrieved from the Swedish Child Health Services register, calculated in autumn 2022 (*N* = 1018) and 2024 (*N* = 1049). The Index is calculated every 2 years through a collaboration between the Register, Statistics Sweden, and all 21 regions in Sweden. We combined the scores from 1018 centres from December 2022 and 1049 centres from November 2024. There were 937 centres included, after we excluded those with less than 50 enrolled children and those that lacked data for either 2022 or 2024 due to opening or closure. The data covered all 21 regions in 2024, but only 20 in 2022, as one region did not participate. Each Child Healthcare Centre was analysed to calculate the difference in the overall Index scores. To identify changes of impact for clinical practice or resource allocation, we used a threshold of more than 0.2 difference in Index value as the cut‐off. In addition, we used the four categories described by Wennergren et al. 2025 [5] to assess whether the healthcare centres changed category between the two time points.

The results showed that the Child Adjusted Care Need Index Category group did not change for 88% of the Child Healthcare Centres. In addition, the change in Index value was no more than 0.2 in 79% of cases. Table 1 shows the most stable categories. We found that 92% of the centres in group one in 2022, which comprised the lower Index values, were still in the same group in 2024. The figure for group four, which comprised the higher Index values, was 96%. There were higher proportions of centres in groups two and three that moved into other categories, and these were 16% and 25% respectively. However, these Index values indicated only small changes in proportions of children with health‐related risk factors. Overall, 133 Child Healthcare Centres changed their Index value by more than 0.2, but only 36 of them switched groups. This study was unable to obtain data on resource allocation and therefore could not estimate the impact of these changes. However, since only 3.8% of all Child Healthcare Centres in this sample both had a large change in Index value and switched groups, the overall impact on resource allocation at the population level may be limited.

**TABLE 1:** Comparison of the number of healthcare centres in each Child Adjusted Care Need Index group between 2022 and 2024, with values per category shown in parentheses.

Child adjusted care need index category group by score	*n* (%) in group 2022	*n* (%) in group 2024	*n* (%) stayed in the same group	*n* (%) moved to group 1	*n* (%) moved to group 2	*n* (%) moved to group 3	*n* (%) moved to group 4
Group 1: (0.00–0.90)	396 (42%)	394 (42%)	364 (92%)	n/a	32 (8%)	0	0
Group 2: (0.91–1.40)	312 (33%)	311 (33%)	263 (84%)	30 (10%)	n/a	19 (6%)	0
Group 3: (1.41–1.99)	116 (12%)	111 (12%)	87 (75%)	0	16 (14%)	n/a	13 (11%)
Group 4: (2.00+)	113 (13%)	121 (13%)	108 (96%)	0	0	5 (4%)	n/a
Total	937	937	822 (88%)	30 (3%)	48 (5%)	24 (3%)	13 (1%)

This short comparison showed that the Index values were quite stable over time and can be used to compare sociodemographic contexts in Swedish Child Health Services. Whether the Index is the optimal way of measuring the sociodemographic impact on children's health is debatable. However, it is well known that the sociodemographic conditions that children grow up in impact both their use of healthcare resources and their health outcomes. The stability observed in this analysis is not just important for the Index itself, but for the broader use of sociodemographic background variables in child health research. Based on this study, we conclude that such variables can be reliably used in studies of child health and service use. This is because they demonstrate a consistent pattern over time, even if the number of Child Healthcare Centres shifts marginally between measurement points.

## Author Contributions

**Rajna Knez:** conceptualization, writing – original draft, supervision. **Anna Grimby‐Ekman:** supervision, writing – review and editing, conceptualization, writing – original draft. **Sofia Dalemo:** supervision, conceptualization, writing – original draft, funding acquisition, writing – review and editing. **Anna Fäldt:** supervision, conceptualization, writing – original draft, writing – review and editing, methodology. **Mattias Wennergren:** conceptualization, investigation, writing – original draft, methodology, visualization, writing – review and editing, software, formal analysis.

## Funding

This project was funded by the Local Research and Development Council Gothenburg and Södra Bohuslän (VGFOUGSB‐976615).

## Conflicts of Interest

The authors declare no conflicts of interest.

## Data Availability

The data that support the findings of this study are available from the corresponding author upon reasonable request.
